# Absence of *Rnf126* causes male infertility with multiple morphological abnormalities of the sperm flagella

**DOI:** 10.1038/s41420-025-02432-w

**Published:** 2025-05-23

**Authors:** Shengnan Wang, Zihan Qin, Juan Liu, Jie Liu, Qiaohua Xiong, Zexiao Wei, Li Wang, Yuming Cao

**Affiliations:** 1 Department of Obstetrics and Gynecology, Perinatal Medical Center, The Fifth Affiliated Hospital of Sun Yat-sen University, Zhuhai, PR China; 2 Department of Obstetrics and Gynecology, Perinatal Medical Center, Zhongnan Hospital of Wuhan University, Wuhan, Hubei China

**Keywords:** Spermatogenesis, Gene expression, Diagnostic markers

## Abstract

Male infertility is primarily caused by impaired flagella development, reduced sperm count, and decreased motility. Despite the involvement of many genes in spermatogenesis, the precise processes remain unclear. The critical E3 ubiquitin ligase *Rnf126* regulates essential cellular processes through ubiquitination-induced protein degradation. It plays a significant role in DNA repair, immune response, and signaling cascades, underscoring its central importance in maintaining cellular homeostasis. However, the mechanisms by which *Rnf126* controls spermatogenesis are not fully understood. This research identifies *Rnf126* as a crucial component in sperm flagellar biogenesis and germ cell development. Through genetic lineage tracing, we show that RNF126 is highly expressed in sperm cells and weakly expressed in Sertoli cells. The germ epithelium of RNF126 deficiencies is characterized by a loss of germ cells due to an increase in germ cell apoptosis at various stages of development, which ultimately results in vesiculation of the spermatogenic tubule. Targeting *Rnf126* results in different types of germ cells reduction, infertility, and microtubule-associated motor activity failure (MMAF), characterized by spermatozoa with truncated, twisted, and malformed flagella. Detailed ultrastructural studies reveal the extent of flagellar damage in the absence of *Rnf126*, highlighting its critical role in maintaining flagellar stability. An important finding is the interaction between RNF126 and BAG6, which regulates sperm synthesis and germ cell development. Clinically, reduced RNF126 levels in sperm from individuals with oligoasthenoteratospermia are significantly different from those in fertile individuals. Investigating *Rnf126* function in spermatogenesis, together with empirical findings on MMAF presentation, may improve our understanding of the developmental processes involved in sperm flagellum formation and contribute to elucidating the causes of male infertility.

## Introduction

Male infertility affects approximately 15% of couples attempting conception, representing a major global health concern [1]. Understanding the complex nature of male infertility is crucial for developing effective support systems and interventions [2]. Male infertility can be categorized into three primary types: azoospermia, teratozoospermia, asthenozoospermia, and oligospermia [3]. The motility and morphology of sperm are vital factors in the intricate equation determining male fertility [4]. These characteristics are essential for assessing oocyte fertilization rates and predicting the likelihood of successful pregnancy. Deficiencies in either parameter can significantly influence male fertility [5]. A substantial subset of male infertility cases, characterized by distinct changes in sperm tail structures, is associated with MMAF [6]. While male infertility has numerous etiological causes, genetic mutations are believed to account for approximately 60% of cases of male-mating infertility [7]. This discovery emphasizes the critical role of genetics in understanding and potentially treating this specific form of infertility.

MMAF constitutes a severe category of sperm defects that significantly affect male fertility [8]. Affected spermatozoa may exhibit abnormalities in their flagellar structure, including shortened, coiled, absent, or irregularly formed flagella, resulting in compromised motility and impaired function [9]. MMAF represents more than mere surface defects, it reflects profound ultrastructural irregularities that can be detected using transmission electron microscopy (TEM) at the nanoscopic scale [10]. These anomalies frequently involve axonemal defects, as well as the absence or deformity of fibrous sheaths (FS) and outer dense fibers (ODFs), which are critical components regulating sperm motility and function [11]. The scientific challenges associated with understanding the genetic basis of flagellar abnormalities in human spermatozoa are substantial [12]. To overcome the limitations of studying sperm developmental defects in humans, the scientific community has developed mutant mouse models to elucidate the genetic and molecular mechanisms of spermatogenesis. Numerous genes, including AKAP3, AKAP4, AK7, CCDC39, DNAH1, CFAP43, CFAP44, CFAP69, CFAP65, CFAP70, CFAP251, and SPATA6, have been implicated in abnormal sperm flagella [13, 14]. While considerable insights into the complexities of spermatogenesis have been gained through the study of genetically modified mouse models, many questions remain unanswered.

RNF126 is an intriguing member of the E3 ubiquitin ligase family, playing multifaceted roles in various biological processes [15]. Encoded by the *Rnf126* gene located on chromosome 7q34, it is essential for attaching ubiquitin molecules to proteins, marking them for degradation [16]. This process affects a wide range of cellular functions, including immune response modulation, DNA repair, and signal transduction [17]. The enzymatic activity of *RNF126* is facilitated by the presence of a conserved ring finger domain, a feature characteristic of E3 ubiquitin ligases. Through this domain, RNF126 can interact with E2 ubiquitin-conjugating enzymes, enabling the transfer of ubiquitin to target proteins, often leading to their degradation via the proteasome [18]. RNF126 is closely linked to the occurrence and progression of various diseases, including breast cancer, brain tumors [19], liver cancer, and others. Studies on RNF126 in triple-negative breast cancer (TNBC) show that it enhances DNA exonuclease activity by physically binding to the MRE11-RAD50-NBS1 (MRN) complex and ubiquitinating MRE11 at specific amino acid sites, thereby promoting homologous recombination repair (HR) and maintaining genome stability [20]. Additionally, RNF126 affects the sensitivity of radiation therapy. Furthermore, RNF126 has been shown to influence the subcellular localization of ferroptosis suppressor protein 1 (FSP1) by interacting with and ubiquitinating FSP1, which in turn regulates the CoQ/CoQH2 ratio, preventing ferroptosis and phospholipid peroxidation [21]. Additionally, by ubiquitinating and degrading liver kinase B1 (LKB1), RNF126 destabilizes this protein, promoting stem-like characteristics, migration, and angiogenesis in hepatocellular carcinoma (HCC) [22]. Accumulating evidence linking RNF126 to various diseases underscores its critical role in maintaining cellular homeostasis.

In this investigation, we identified a deleterious variation of *Rnf126* that results in male infertility and asthenoteratozoospermia. By generating *Rnf126* gene-knockout mice, we elucidated the crucial role of the *Rnf126* gene in germ cell development and, consequently, male fertility. The observed abnormalities in sperm motility and flagellar development highlight the essential role of *RNF126* in the complex process of spermatogenesis. The interaction between BAG6 and RNF126 influences BAG6 expression. This novel finding provides insight into the observed increase in germ cell apoptosis and abnormalities in sperm flagellar formation in mice lacking the *Rnf126* gene. Our research suggests that *Rnf126* defects may serve as both a pathogenic factor and a diagnostic marker for male infertility in cases of asthenoteratozoospermia.

## Result

### RNF126 is highly expressed and localized specifically during spermatogenesis

**Fig. 1:**
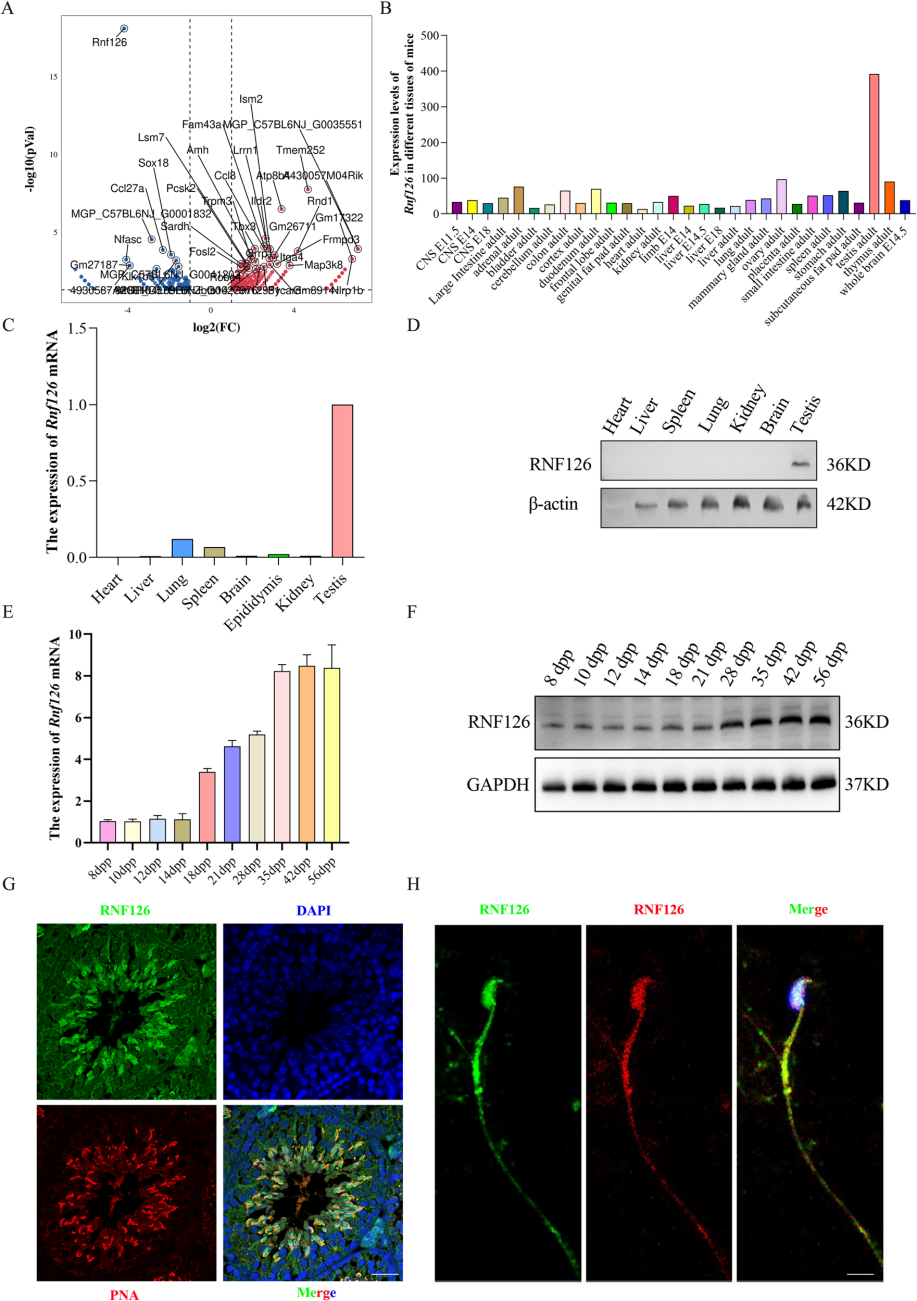
Expression profile and specific localization of RNF126. **A** Differential *RNF126* expression in normal vs. teratospermia sperm. When comparing sperm from oligoasthenoteratospermia patients with normal sperm, RNA sequencing analysis shows that the *RNF126* expression of the pathological group is significantly lower. **B** NCBI database analysis shows that the expression level of *Rnf126* in the testis is significantly higher than in other organs. **C** qRT-PCR assays confirm the highest expression of *Rnf126* mRNA in testis samples of all tissues analyzed, including heart, liver, spleen, lung, kidney and brain (*n* = 3). **D** Western blotting is used to validate the protein expression levels of RNF126 and reveals significant enrichment in the testis when compared to other tissues examined, such as the heart, liver, spleen, lung, kidney, and brain. The control utilized was β-actin. **E** *Rnf126* mRNA expression was detected by qRT-PCR during several testicular developmental stages (postnatal day 8–56), and the results show significant increases during the spermatogenesis stages, (*n* = 3). **F** Western blot is used to check the expression dynamics of RNF126 protein at different postnatal stages of testicular development. A significant increase is detected during spermiogenesis, with GAPDH acting as a charge control. **G** Using PNA (red) and DAPI (blue) for additional labeling, immunofluorescence images demonstrate the precise location of RNF126 (green) within spermatogenic cells during late spermiogenesis. The scale bar is 50 μm. **H** Mature spermatozoa were removed from the cauda epididymitis of 56-day-old mice, and immunofluorescence staining was used to show the specific localization of RNF126 (green: rabbit, red: mouse) within the sperm heads and flagella. A blue stain called DAPI is applied to the nuclei. 50 μm is the scale bar’s size.

RNA sequencing revealed that sperm samples from oligoasthenoteratozoospermia (OAT) patients exhibited significantly lower *RNF126* expression levels compared to those from normozoospermic individuals (Fig. 1A). Data from the NCBI database indicated that *RNF126* expression in testicular tissue was markedly higher than in other organs (Fig. 1B). This observation was further validated by reverse transcription polymerase chain reaction (RT-PCR) and western blot analyses, which consistently demonstrated elevated *Rnf126* expression in the testes relative to major organs, including the heart, liver, brain, and kidneys (Fig. 1C, D).

To investigate temporal changes in RNF126 expression during mammalian gametogenesis, we observed a significant upregulation of RNF126 in the post-spermatogenesis stage (Fig. 1E, F), a period critical for the development of sperm viability and motility. The expression pattern of RNF126 was consistent in both humans and mice (Fig. S1A). Immunofluorescence analyses and comparative verification of staining results of *Rnf126*^−/−^ revealed a strong RNF126 signal in spermatocytes undergoing spermiogenesis within testicular sections (Figs. 1G and S1B). Extending the analysis to mature sperm in the epididymal tail, RNF126 was distinctly localized to the sperm head and flagellum (Fig. 1H). These findings suggest that RNF126 plays a crucial role in maintaining the structural integrity and functionality of these sperm components.

### RNF126 is essential for male germ cell development and spermatogenesis

**Fig. 2:**
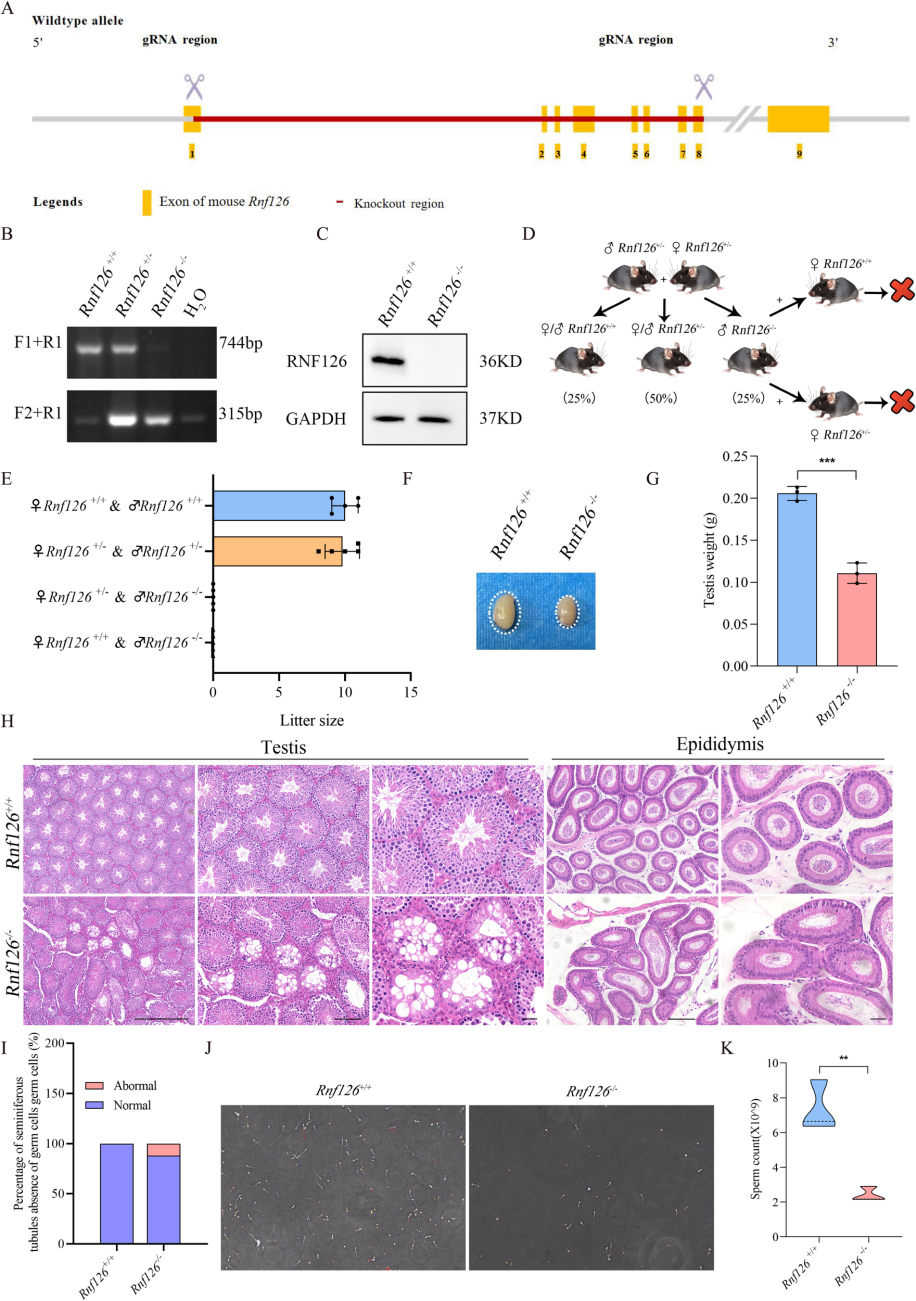
RNF126 is essential for germ cell development and sperm production. **A** Diagram showing how the targeted disruption approach was used to produce mice lacking *Rnf126*. **B** PCR analysis to determine the genotypes of *Rnf126* mice that are wild type (*Rnf126*^*+/+*^), heterozygous (*Rnf126*^+/−^) and homozygous knockout (*Rnf126*^−/−^). When primer sets F1 + R1 are amplified, a 744-bp fragment typical of *Rnf126*^*+/+*^ mice is produced, while with F2 + R1 a 315-bp product indicative of *Rnf126*^−/−^ mice is amplified. Heterozygosity is indicated by the simultaneous detection of both fragments. Water acts as a negative control. **C** Testis tissue extracts from *Rnf126*^*+/+*^ and *Rnf126*^−/−^ mice were subjected to Western blot analysis, which confirmed the successful deletion of the gene by showing that the RNF126 protein band was completely absent in the knockout samples. **D** An overview of mating strategies between different genotypes, emphasizing the infertile phenotype of *Rnf126*^−/−^ males. **E** Quantitative analysis of **D**, emphasizing the infertility associated with the absence of RNF126 in males (*n* = 5). Data are presented as mean ± S.D. Student’s *t* test; ***p < 0.001. **F** Comparative images of testes from *Rnf126*^*+/+*^ and *Rnf126*^−/−^ mice show a significant reduction in the size of the *Rnf126*^−/−^ testes. **G** The testis weights of *Rnf126*^*+/+*^ and *Rnf126*^−/−^ mice were compared; the results showed a significant decrease in *Rnf126*^*+/+*^ (*p* < 0.001) (*n* = 3). Data are presented as mean ± S.D. Student’s *t* test; ***p < 0.001. **H** Hematoxylin and eosin (H&E) stained histological sections of testes (scale bars: left = 200 μm, middle = 100 μm, right = 50 μm) and epididymis (scale bars: left = 100 μm, right = 50 μm) from *Rnf126*^*+/+*^ and *Rnf126*^−/−^ Mice displaying structural changes associated with *Rnf126* deficiency. **I** The proportion of seminiferous tubules with a significant absence of germ cells in the testicular sections of *Rnf126*^*+/+*^ and *Rnf126*^−/−^ mice. Data are presented as average percentage, *n* = 3 mice for each group, and 100 tubules were counted for each mouse. **J** Examination of sperm motility and number in *Rnf126*^*+/+*^ compared to and *Rnf126*^−/−^. Sperm motility and the number of *Rnf126* knockout animals decreased significantly. **K** Statistical examination of **I** demonstrates that *Rnf126* deficiency negatively impacts sperm motility and count in *Rnf126*^−/−^ mice. (*n* = 6). Data are presented as mean ± S.D. Student’s *t* test; **p < 0.01.

The *Rnf126* knockout mouse model (C57BL/6J) was developed in collaboration with Cypage Biotechnology using CRISPR/Cas-mediated genomic engineering. Exons 1–8 of the *Rnf126* gene were targeted, resulting in the deletion of a 5988 bp region (Fig. 2A). Following polymerase chain reaction (PCR) amplification, the genotypes were identified via agarose gel electrophoresis. Each genotype exhibited a distinct banding pattern: *Rnf126*^*+/+*^ (wild type) showed only the WT fragment (744 bp), *Rnf126*^−/−^ (knockout) showed only the KO fragment (315 bp), and heterozygous (*Rnf126*^+/−^) samples displayed both WT (744 bp) and KO (315 bp) fragments. Band sizes were compared against a molecular weight ladder to ensure accurate identification of genotypes (Fig. 2B). Western blot analyses confirmed the absence of RNF126 protein in *Rnf126*^−/−^ mice, as no detectable bands corresponding to RNF126 were observed. In contrast, robust bands were evident in *Rnf126*^*+/+*^ samples, confirming complete loss of RNF126 protein expression in the knockout mice (Fig. 2C). Male *Rnf126*^−/−^ mice exhibited profound infertility, as they failed to produce offspring despite regular and prolonged mating with fertile females (Fig. 2D, E). Further analysis of *Rnf126*^−/−^ testes revealed significantly smaller dimensions and reduced testicular weight compared to those of *Rnf126*^*+/+*^ mice (Fig. 2F, G). Hematoxylin and eosin (HE) staining demonstrated a striking absence of germ cells in 12% of seminiferous tubules of *Rnf126*^−/−^ mice, indicative of impaired germ cell development (Fig. 2H, I). Additionally, the sperm reservoir in the epididymis was drastically depleted in *Rnf126*^−/−^ mice (Figs. 2J, K and S1C). These findings underscore the essential role of RNF126 in maintaining the normal functioning of the male reproductive system, particularly in processes such as sperm development, maturation, and viability.

### RNF126 as a pivotal factor in the dynamic landscape of reproductive development

**Fig. 3:**
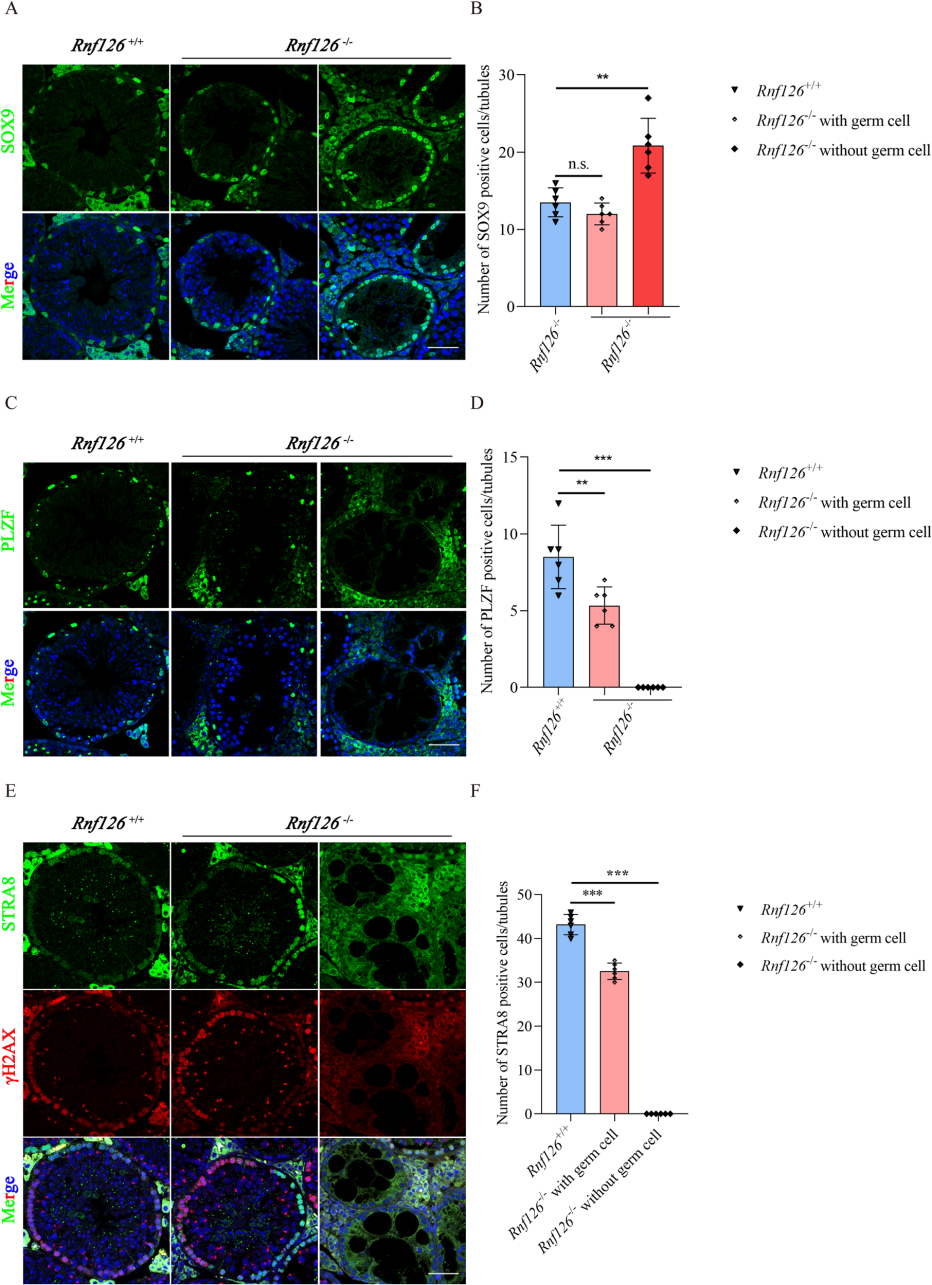
Altered composition of Sertoli cells and spermatogonium in *Rnf126*^−/−^ mice. **A** Immunofluorescence staining for SOX9, a marker for Sertoli cells, in seminiferous tubules of *Rnf126*^*+/+*^ and *Rnf126*^−/−^ mice. In the absence of germ cells, *Rnf126*^−/−^ testes have a higher number of SOX9-positive cells. Scale bar = 50 μm. **B** Quantitative analysis of SOX9-positive cells in (**A**) (*n* = 6). Data are presented as mean ± S.D. Student’s *t* test; **p < 0.01. **C** Immunofluorescence labeling for PLZF, a marker for undifferentiated spermatogonia, in seminiferous tubules of *Rnf126*^*+/+*^ and *Rnf126*^−/−^ mice. In both the presence and absence of germ cells, *Rnf126*^−/−^ seminiferous tubules exhibit reduced numbers of PLZF-positive cells. Scale bar = 50 μm. **D** Quantitative analysis of PLZF-positive cells in (**C**) (*n* = 6). Data are presented as mean ± S.D. Student’s *t* test; ***p < 0.001, **p < 0.01. **E** Immunostaining for STRA8, a marker for preleptotene spermatocytes, in seminiferous tubules of *Rnf126*^*+/+*^ and *Rnf126*^−/−^ mice. Regardless of the presence of germ cells, the number of STRA8-positive cells is significantly lower in the *Rnf126*^−/−^ seminiferous tubules. Scale bar = 50 μm. **F** Quantitative analysis of STRA8-positive cells in (**E**) (*n* = 6). Data are presented as mean ± S.D. Student’s *t* test; ***p < 0.001.

**Fig. 4:**
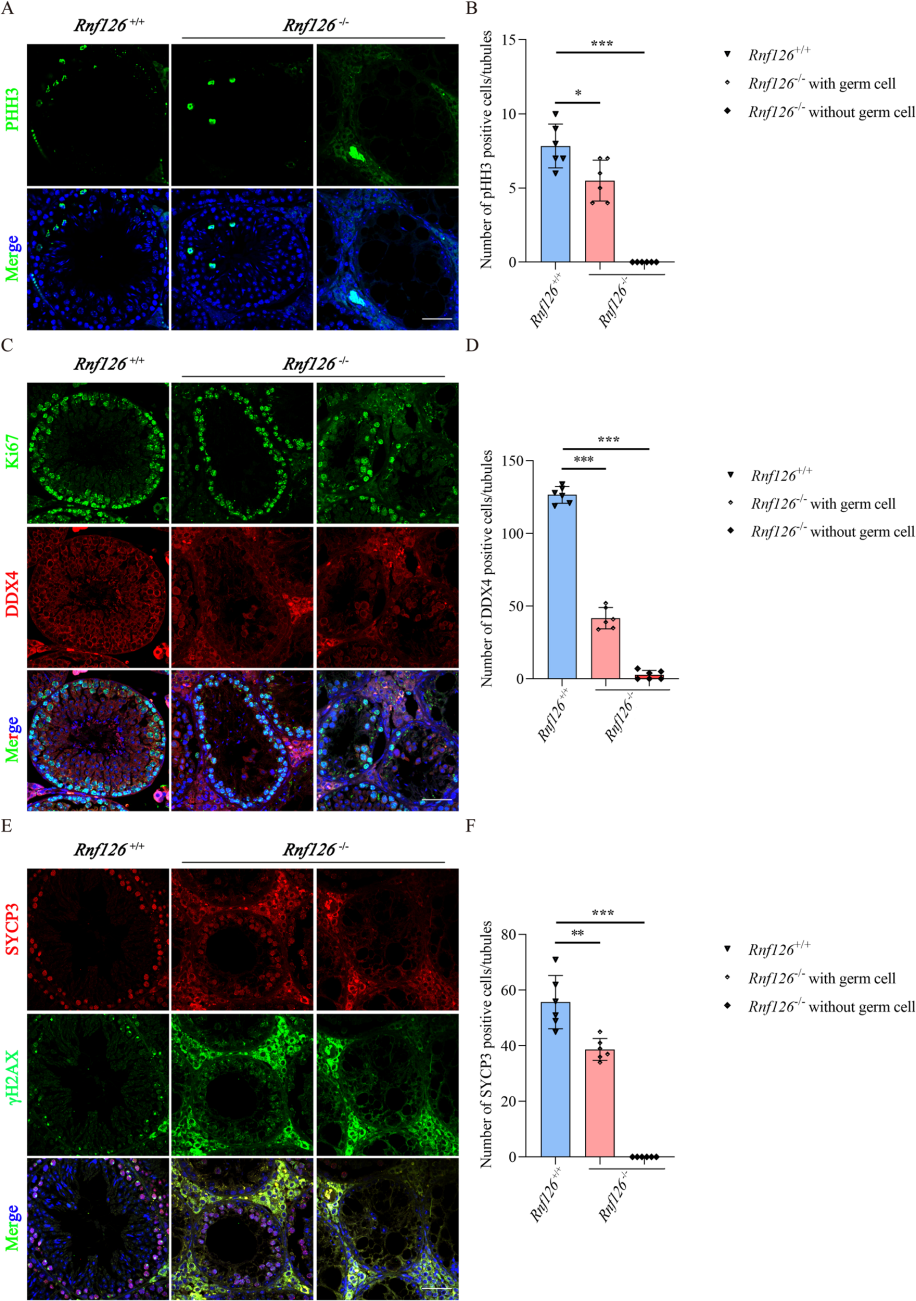
Decreased germ cells in mice lacking the *Rnf126* gene. **A** Immunofluorescence staining for PHH3 in seminiferous tubules of *Rnf126*^*+/+*^ and *Rnf126*^−/−^ mice. The number of PHH3-positive cells appears to have increased in *Rnf126*^−/−^ tests lacking germ cells. Scale bar = 50 μm. **B** Quantitative analysis of PHH3-positive cells in (**A**) (*n* = 6). Data are presented as mean ± S.D. Student’s *t* test; ***p < 0.001, *p < 0.05. **C** Immunostaining for DDX4 in seminiferous tubules of *Rnf126*^*+/+*^ and *Rnf126*^−/−^ mice. The number of DDX4-positive cells decreases dramatically in germ cell-depleted *Rnf126* KO testes. Scale bar = 50 μm. **D** Quantitative analysis of DDX4-positive cells in (**C**), (*n* = 6). Data are presented as mean ± S.D. Student’s *t* test; ***p < 0.001. **E** Immunofluorescence labeling for SYCP3 in seminiferous tubules of *Rnf126*^*+/+*^ and *Rnf126*^−/−^ mice. The SYCP3-positive cell number also decreases significantly in germ cell-less *Rnf126*^−/−^ testes. Scale bar = 50 μm. **F** Quantitative analysis of SYCP3-positive cells in (**E**), (*n* = 6). Data are presented as mean ± S.D. Student’s *t* test; ***p < 0.001, **p < 0.01.

To elucidate the critical role of *RNF126* in germ cell development, we conducted a comprehensive study employing specific markers that delineate distinct stages of germ cell maturation. SOX9 (SRY-Box 9) marks Sertoli cells, the stromal cells that provide a supportive microenvironment crucial for spermatogenesis [23]. Using a comparative histological approach with SOX9 immunofluorescence, we assessed Sertoli cell populations in *Rnf126*^−/−^ and *Rnf126*^*+/+*^ testes. Although the number of Sertoli cells per unit area in regions containing germ cells did not differ significantly between *Rnf126*^−/−^ and *Rnf126*^*+/+*^ mice, a marked increase in Sertoli cell density was observed in seminiferous tubules devoid of germ cells in *Rnf126*^−/−^ testes (Fig. 3A, B). PLZF (Promyelocytic Leukemia Zinc Finger) identifies undifferentiated spermatogonia, the foundational germ cells that give rise to spermatogonia stem cells [24]. PLZF staining of testicular sections revealed a significant reduction in the number of PLZF-positive cells in both germ cell-containing and germ cell-deficient tubules in *Rnf126*^−/−^ mice compared to *Rnf126*^*+/+*^ controls (Fig. 3C, D). STRA8 (Stimulated by Retinoic Acid Gene 8) is expressed in spermatogonia entering meiosis, marking the transition from mitotic proliferation to meiotic division characteristic of spermatogenesis [25]. Analysis showed a substantial decrease in STRA8-positive spermatogonia in *Rnf126*^−/−^ tubules, even in those containing germ cells, compared to *Rnf126*^*+/+*^ samples (Fig. 3E, F). PHH3 (Phosphorylated Histone H3) serves as an indicator of active cell division, particularly during metaphase and anaphase [26]. In *Rnf126*^−/−^ seminiferous tubules, PHH3-positive cells were significantly reduced, even in regions containing germ cells, relative to *Rnf126*^*+/+*^ (Fig. 4A, B). DDX4 (Vasa), a DEAD box RNA helicase, is a universal marker for germ cells essential for germ cell identity and spermatogenesis [27]. *Rnf126*^−/−^ mice exhibited a pronounced reduction in the number of DDX4-positive cells, even in seminiferous tubules presumed to contain germ cells, compared to controls (Fig. 4C, D). SYCP3 (Synaptonemal Complex Protein 3) specifically highlights primary spermatocytes undergoing meiosis I and is critical for synaptonemal complex formation [28]. A striking reduction in SYCP3-positive cells was observed in the seminiferous tubules of *Rnf126*^−/−^ mice compared to *Rnf126*^*+/+*^, even in regions containing germ cells (Fig. 4E, F). This finding suggests that RNF126 deficiency adversely affects meiotic development. The collective data underscores the essential role of RNF126 in maintaining and regulating germ cell development at various stages.

### Synapsis and DNA damage repair are unaffected by the absence of *Rnf126*

**Fig. 5:**
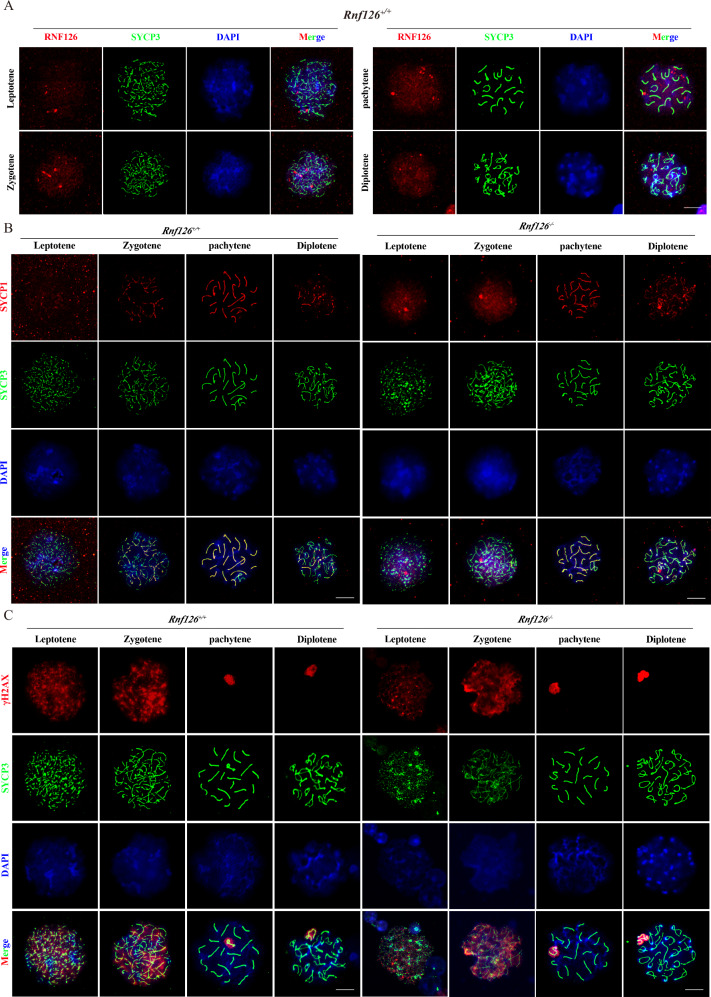
Chromosome synapsis is normal in mice lacking *Rnf126.* **A** Immunofluorescence microscopy shows the localization of RNF126 (red) adjacent to the synaptonemal complex component SYCP3 (green) with DNA stained by DAPI (blue) in *Rnf126*^*+/+*^ and *Rnf126*^−/−^ mice. Scale bar = 50 μm. **B** Immunofluorescence staining for SYCP1 (red), another synaptonemal complex component, and SYCP3 (green), in *Rnf126*^*+/+*^ and *Rnf126*^−/−^ mice, with DAPI-stained DNA shown in blue. Scale bar = 50 μm. **C** Immunofluorescence imaging of γH2AX (red), SYCP3 (green) and DAPI-stained DNA (blue) in spermatocytes in *Rnf126*^*+/+*^ and *Rnf126*^−/−^ mice. Scale bar = 50 μm.

To further investigate the role of RNF126 in spermatocyte meiosis, we examined its localization during spermatocyte development. RNF126 displayed a diffuse distribution in spermatocytes at the zygotene and pachytene stages, with no evidence of concentration around specific structures (Fig. 5A). The synaptonemal complex (SC), composed of proteins such as SYCP1 and SYCP3, facilitates homologous chromosome pairing and synapsis during meiosis. Immunostaining for SYCP1 and SYCP3 was performed to evaluate SC formation in testis sections [29]. Our results showed no significant differences in SC formation between *Rnf126*^−/−^ and *Rnf126*^*+/+*^ spermatocytes (Fig. 5B). To assess the impact of RNF126 on DNA damage repair during spermatogenesis, we performed immunofluorescence staining for γH2AX, a marker of DNA double-strand breaks (DSBs). Comparable levels and spatial distribution of γH2AX were observed in spermatocytes from *Rnf126*^−/−^ and *Rnf126*^*+/+*^ mice (Fig. 5C). These observations suggest that RNF126 does not significantly influence synapsis or DNA damage repair in spermatocytes, prompting speculation regarding alternative functions of RNF126 in spermatogenesis.

**Fig. 6:**
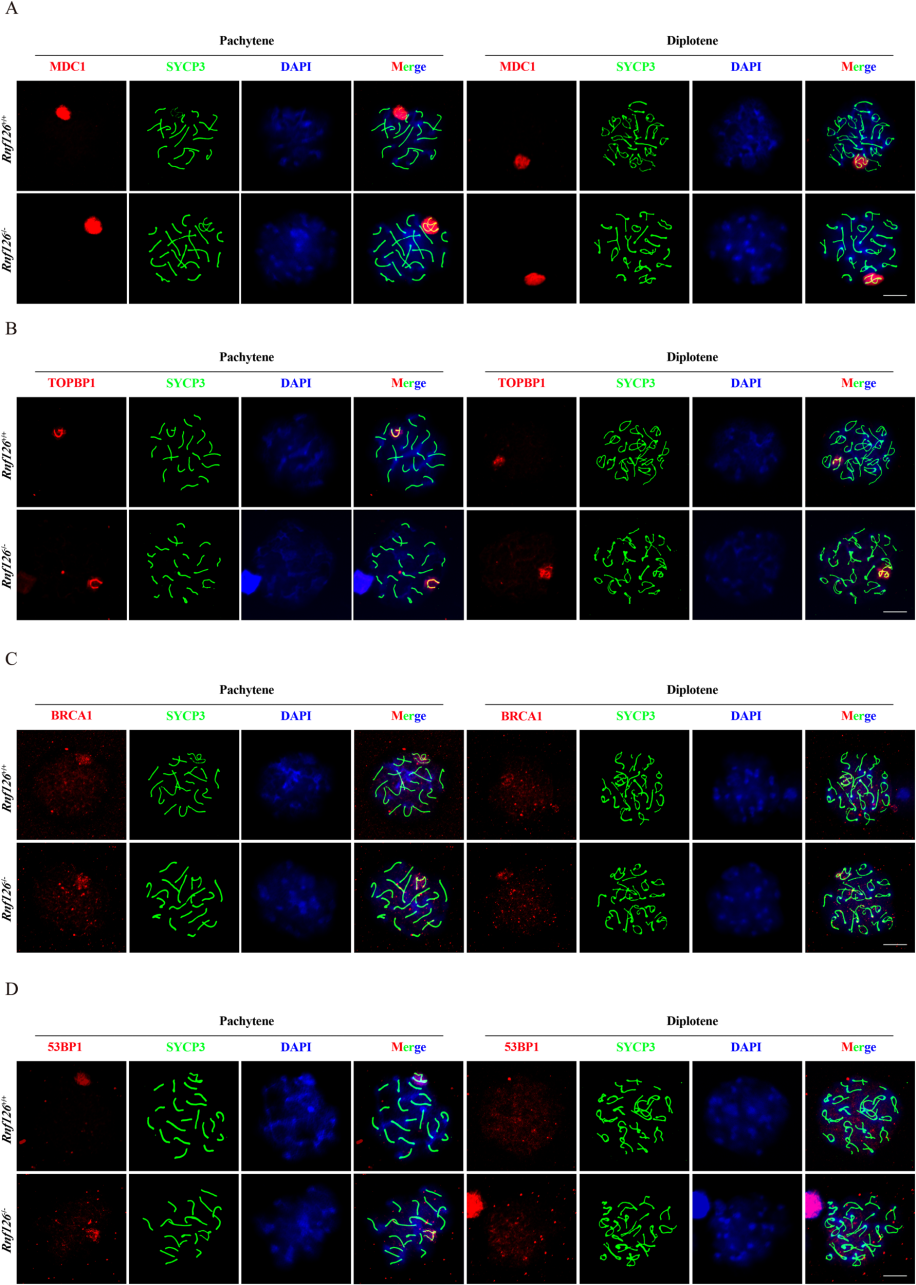
Unaltered DNA damage repair in *Rnf126*^−/−^ mice. **A** Immunofluorescence of MDC1 (red), SYCP3 (green), and DAPI (blue) in *Rnf126*^*+/+*^ and *Rnf126*^−/−^ pachytene spermatocytes. Scale bar = 50 μm. **B** Immunofluorescence of TOPBP1 (red), SYCP3 (green) and DAPI (blue) in *Rnf126*^*+/+*^ and *Rnf126*^−/−^ pachytene spermatocytes. Scale bar = 50 μm. **C** Immunofluorescence of BRCA1 (red), SYCP3 (green) and DAPI (blue) in *Rnf126*^*+/+*^ and *Rnf126*^−/−^ pachytene spermatocytes. Scale bar = 50 μm. **D** Immunofluorescence of 53BP1 (red), SYCP3 (green) and DAPI (blue) in *Rnf126*^*+/+*^ and *Rnf126*^−/−^ pachytene spermatocytes. Scale bar = 50 μm.

Maintaining genomic integrity during critical developmental stages, such as meiosis, requires efficient DNA damage repair. MDC1 (Mediator of DNA Damage Checkpoint 1) plays a pivotal role in the initial response to DNA double-strand breaks (DSBs) during the meiotic process [30]. To investigate whether RNF126 influences MDC1 localization, spermatocytes from *Rnf126*^*+/+*^ and *Rnf126*^−/−^ mice were isolated and analyzed during the pachytene and diplotene stages of meiosis. The analysis demonstrated that MDC1 localization patterns were similar in both *Rnf126*^*+/+*^ and *Rnf126*^*−/−*^ spermatocytes at these stages, indicating that RNF126 is not required for MDC1’s initial recruitment to DSBs during meiosis (Fig. 6A). TOPBP1 (Topoisomerase II Binding Protein 1), a critical mediator in the DNA damage response (DDR) pathway, is essential for checkpoint activation and repair processes [31]. We visualized and compared the localization of TOPBP1 in spermatocytes from *Rnf126*^*+/+*^ and *Rnf126*^−/−^ mice. The analysis revealed no discernible differences in the density, distribution, or morphology of TOPBP1 signals between the genotypes, suggesting that RNF126 does not directly regulate the spatial organization of TOPBP1 during meiotic DNA repair (Fig. 6B). BRCA1, a cornerstone protein in the DDR, plays a central role in maintaining genomic stability. Examination of BRCA1 localization in spermatocytes from *Rnf126*^*+/+*^ and *Rnf126*^−/−^ mice showed similar patterns at the pachytene and diplotene stages (Fig. 6C). 53BP1 (p53 Binding Protein 1), a key regulator in the DDR pathway involved in DNA damage detection and response [32], was also analyzed. The localization of 53BP1 in spermatocytes derived from *Rnf126*^*+/+*^ and *Rnf126*^−/−^ mice was found to be comparable during the pachytene and diplotene stages of meiotic prophase I (Fig. 6D). These findings provide robust evidence that the absence of RNF126 does not affect the localization of MDC1, TOPBP1, BRCA1, or 53BP1 during meiosis. However, RNF126 may play other roles in the DDR that are not reflected in altered localization patterns.

### *Rnf126* deficiency induces sperm head and tail malformations

**Fig. 7:**
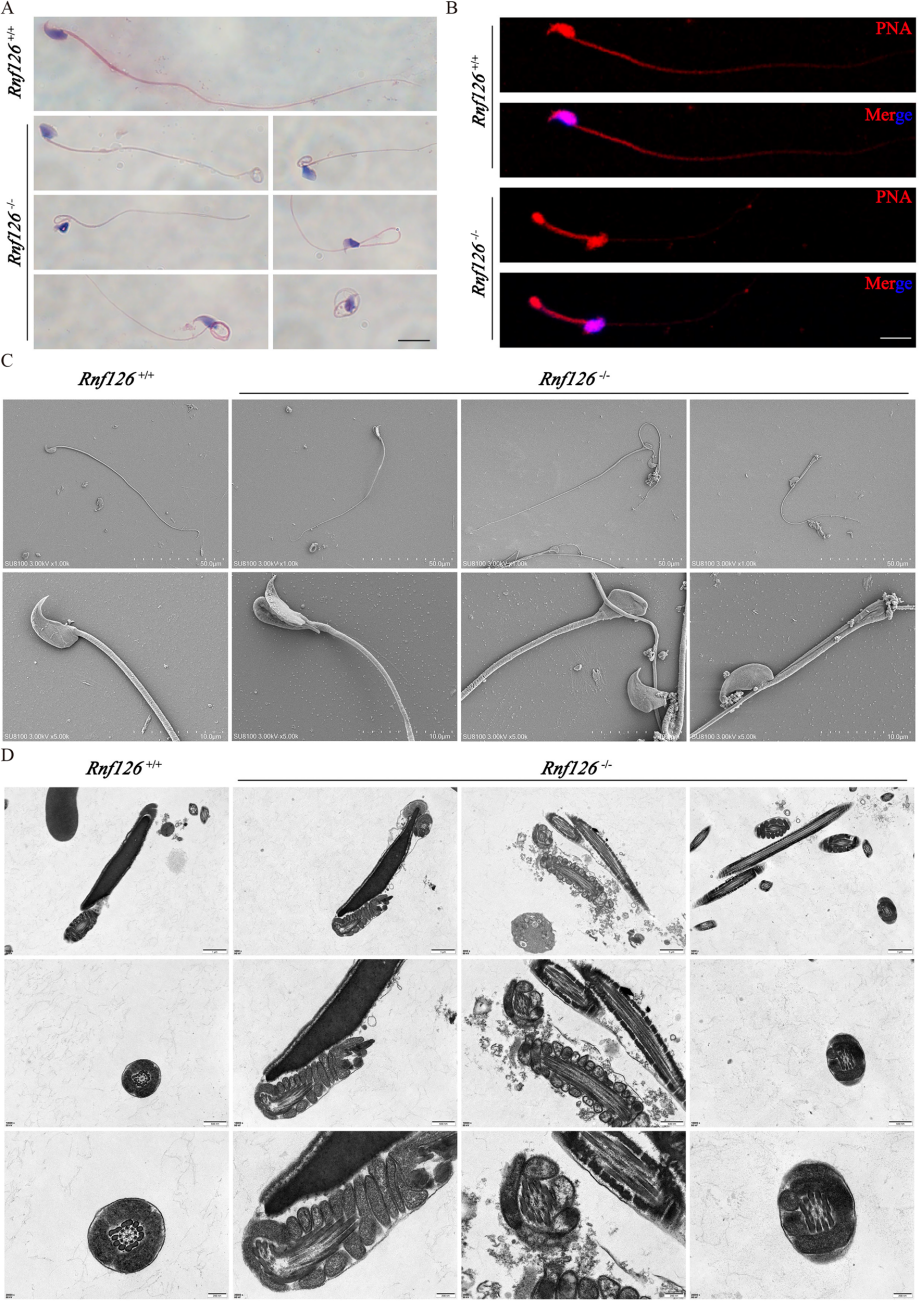
Abnormalities in sperm morphology from *Rnf126*^−/−^ mice. **A** Hematoxylin and eosin staining of sperm between *Rnf126*^*+/+*^ and *Rnf126*^−/−^ mice. Scale bar = 50 μm. **B** Peanut agglutinin fluorescent staining of sperm between *Rnf126*^*+/+*^ and *Rnf126*^−/−^ mice. PNA (red), DAPI (blue), scale bar = 50 μm. **C** Scanning electron microscopic analysis of sperm from *Rnf126*^*+/+*^ and *Rnf126*^−/−^ mice. Scale bar (top) = 50 μm, scale bar (bottom) = 10 μm. **D** Transmission electron microscopy of sperm ultrastructures in *Rnf126*^*+/+*^ and *Rnf126*^−/−^ mice. Scale bar (top) = 1 μm, scale bar (middle) = 500 nm, scale bar (bottom) = 200 nm.

To evaluate the impact of *Rnf126* deficiency on sperm morphology, mature sperm were obtained from the cauda epididymis of *Rnf126*^*+/+*^ and *Rnf126*^−/−^ mice. Hematoxylin and eosin (H&E) staining revealed pronounced abnormalities in the sperm of *Rnf126*^−/−^ mice, including excessive coiling, bending, and fragmentation of sperm tails. Furthermore, a subset of sperm exhibited severe head malformations, deviating significantly from the streamlined, elongated morphology characteristic of normal sperm (Fig. 7A). Consistent with these findings, peanut agglutinin (PNA) staining confirmed significant abnormalities in sperm morphology in *Rnf126*^−/−^ mice (Fig. 7B). In *Rnf126*^−/−^ mice, the percentage of abnormal sperm reached 100% (Fig. S1D). To further investigate these defects, scanning electron microscopy (SEM) was employed to examine the ultrastructure of sperm. SEM analysis revealed extensive structural abnormalities in *Rnf126*^−/−^ sperm, including pronounced tail curling, bending, and kinking, as well as conspicuous head malformations. These abnormalities were absent in *Rnf126*^*+/+*^ sperm (Fig. 7C). Transmission electron microscopy (TEM) provided additional insights into the subcellular defects underlying these morphological abnormalities. TEM analysis of *Rnf126*^−/−^ sperm identified two major areas of concern: defects in axonemal microtubules and alterations in acrosomal structure (Fig. 7D). These findings highlight the critical role of RNF126 in maintaining sperm ultrastructure and functionality. Future studies are warranted to elucidate the molecular mechanisms by which RNF126 contributes to the integrity and functionality of spermatozoa.

### *Rnf126* deficiency disrupts key pathways essential for cell survival

**Fig. 8:**
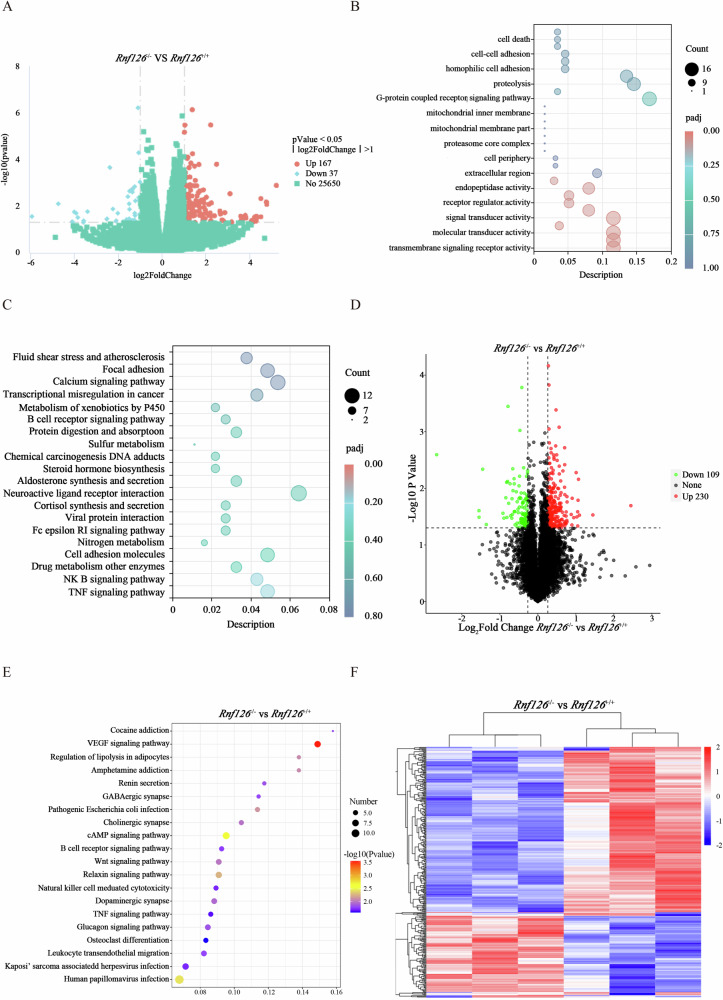
RNF126 plays a crucial role in developmental pathways and cell death regulation. **A** Visualization of differential gene expression between testicular tissues from *Rnf126*^*+/+*^ and *Rnf126*^−/−^ mice by RNA sequencing. **B** GO enrichment analysis of significantly differentially expressed genes identified by RNA-seq contrasts between *Rnf126*^*+/+*^ and *Rnf126*^−/−^ testis tissues. **C** KEGG pathway enrichment analysis of the differentially regulated transcriptome in *Rnf126*^−/−^ testis tissue. **D** Comparison of protein abundance in *Rnf126*^*+/+*^ and *Rnf126*^−/−^ testis tissues using quantitative proteomics. **E** Analysis of KEGG pathway enrichment of proteins exhibiting differential abundance between *Rnf126*^*+/+*^ and *Rnf126*^−/−^ testis tissues. **F** Hierarchical cluster heatmap depicting the comparative expression profiles of proteins significantly modulated in *Rnf126*^−/−^ testis tissues compared to *Rnf126*^*+/+*^.

To elucidate the molecular mechanisms through which RNF126 influences cellular processes, RNA sequencing (RNA-seq) was conducted to compare the transcriptomes of testicular tissues from *Rnf126*^*+/+*^ and *Rnf126*^−/−^ mice. The analysis identified a substantial number of differentially expressed genes (DEGs) in the testes of *Rnf126*^−/−^ mice compared to *Rnf126*^*+/+*^ controls. Specifically, 167 genes were significantly upregulated, while 37 genes were downregulated in *Rnf126*-deficient testes (Fig. 8A). Gene Ontology (GO) term enrichment analysis revealed a strong association of these DEGs with apoptotic processes, indicating that *Rnf126* plays a critical role in regulating cell survival and apoptosis within the germinal epithelium (Fig. 8B). Further investigation using KEGG pathway analysis demonstrated disruption of several key signaling cascades in *Rnf126*^−/−^ mice (Fig. 8C). Extending the analysis to the proteomic level revealed additional insights into the molecular effects of *Rnf126* deficiency (Fig. 8D). Proteomic profiling identified 230 upregulated proteins and 109 downregulated proteins in *Rnf126*-deficient testes compared to *Rnf126*^*+/+*^ controls (Fig. 8E). KEGG pathway enrichment analysis of these proteins highlighted a complex network of signaling pathways influenced by *Rnf126*, underscoring its broad regulatory impact on testis biology (Fig. 8F). The integration of transcriptomic and proteomic data confirms the evidence for *Rnf126* as a central regulator of germ cell development and flagella function and underscores its central role in ensuring the integrity of cell pathways, which are essential for testicular germ cell survival and the formation of sperm flagella.

### RNF126 interaction with BAG6: a synergistic role in germ cell development

**Fig. 9:**
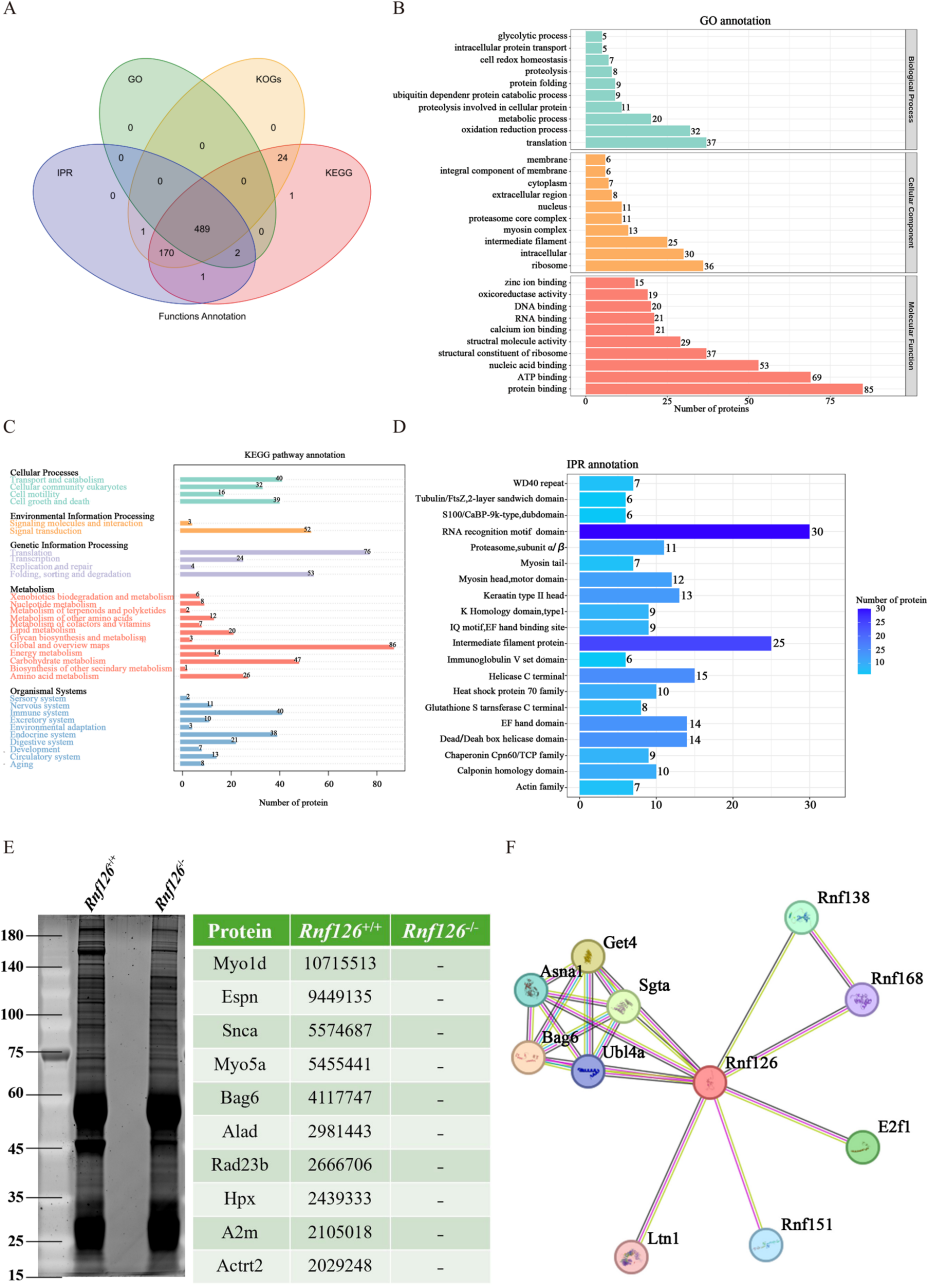
RNF126 co-regulates spermatogenesis through its interaction with BAG6. **A** Venn diagram analysis of proteome profiles obtained from immunoprecipitates of RNF126 in wild-type mouse testis lysates (*Rnf126*^*+/+*^) compared to *Rnf126*^−/−^. **B** Genetic ontological classification of distinct RNF126 immunoprecipitates identified from testis lysates of *Rnf126*^*+/+*^ and *Rnf126*^−/−^ mice. **C** KEGG for RNF126-associated proteins from *Rnf126*^*+/+*^ and *Rnf126*^−/−^ testis lysates. **D** IPR annotations for RNF126-associated proteins from *Rnf126*^*+/+*^ and *Rnf126*^−/−^ testis lysates. **E** Differential abundance of RNF126 immunoprecipitates from *Rnf126*^*+/+*^ compared to *Rnf126*^−/−^ testis lysates. **F** The STRING database’s determination of the protein-protein interaction network centered on RNF126 is shown graphically.

To further investigate the mechanistic role of RNF126 in male fertility and spermatogenesis, immunoprecipitation mass spectrometry (IP-MS) was employed to identify proteins that interact with RNF126 in vivo. Proteomic analysis identified 489 proteins significantly associated with *Rnf126* deficiency (Fig. 9A). GO classification of these proteins revealed a prominent involvement in translation and ribosome biogenesis, suggesting that RNF126 may regulate protein synthesis and ribosome function within the testes (Fig. 9B). KEGG pathway analysis demonstrated the association of these proteins with multiple signaling pathways, including those related to apoptosis and developmental processes, emphasizing RNF126’s potential influence on germ cell survival and differentiation (Fig. 9C). Furthermore, IPR analysis linked these proteins to internal coding regions, suggesting a regulatory role in gene expression, a critical aspect of spermatogenesis (Fig. 9D).

Among the identified proteins, BAG6 emerged as a key factor due to its testis-specific expression and essential role in spermatogenesis (Fig. 9E). Analysis using the STRING database confirmed that RNF126 and BAG6 are components of a shared cellular network (Fig. 9F). Based on these findings, we hypothesize that RNF126 exerts its influence on spermatogenesis and germ cell development, at least in part, through its interaction with BAG6.

**Fig. 10:**
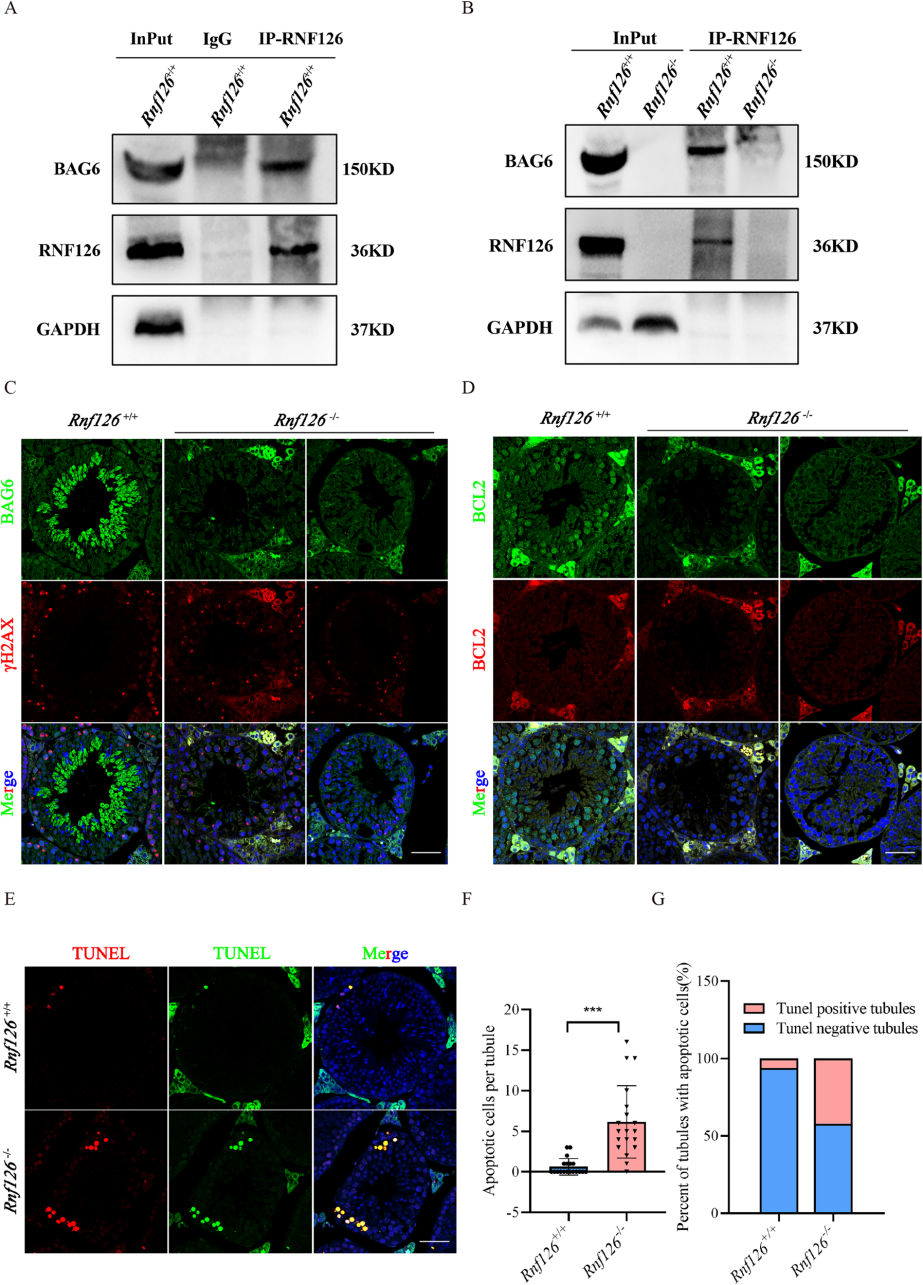
Expression and functional deficiency of BAG6 in *Rnf126*^−/−^. **A** Demonstration of RNF126 and BAG6 interaction via co-immunoprecipitation (Co-IP) assays using testis lysates from *Rnf126*^*+/+*^ mice. The input serves as a control for total protein expression, IgG as a negative control for nonspecific binding, and RNF126-IP confirms the specific interaction between RNF126 and BAG6 under physiological conditions. **B** Verification of RNF126-BAG6 interaction in lysates of *Rnf126*^*+/+*^ and *Rnf126*^−/−^ testes using input and RNF126-IP approaches. **C** Immunofluorescence to examine the location of BCL2 in testis sections from *Rnf126*^*+/+*^ and *Rnf126*^−/−^ mice. γH2AX in turn helps to differentiate between germ cell types. Scale bar = 50 μm. **D** TUNEL staining on testicular tissue sections to compare the level of cell death between *Rnf126*^*+/+*^ and *Rnf126*^−/−^ mice. Scale bar = 50 μm. **E** Quantification of apoptotic cells in individual seminiferous tubules of *Rnf126*^*+/+*^ and *Rnf126*^−/−^ testis tissue sections. Twenty seminiferous tubules per group were carefully examined. **F** Quantitative analysis of seminiferous tubules showing positive indicators of apoptosis in *Rnf126*^*+/+*^ and *Rnf126*^−/−^ testis tissue sections. Each group randomly counted 20 spermatogenic tubules from 3 different mice. Data are presented as mean ± S.D. Student’s *t* test; ***p < 0.001. **G** Spermatogenic tubule counts of TUNEL-positive cells in mice of both *Rnf126*^*+/+*^ and *Rnf126*^−/−^ exist.

Co-immunoprecipitation (Co-IP) experiments were conducted to validate the physical interaction between RNF126 and BAG6 under physiological conditions. Using an RNF126-specific antibody in Co-IP assays with *Rnf126*^*+/+*^ testicular extracts, bands corresponding to RNF126 and BAG6 were successfully detected. In contrast, control assays utilizing IgG antibodies in place of the RNF126-specific antibody did not reveal any bands corresponding to RNF126 or BAG6, confirming the specificity of the interaction (Fig. 10A). Consistent with this finding, Co-IP experiments performed on *Rnf126*^−/−^ testicular extracts did not detect any bands corresponding to RNF126 or BAG6, even when the RNF126-specific antibody was employed, thereby establishing that the interaction between these two proteins is strictly dependent on the presence of RNF126 (Fig. 10B). Immunofluorescence microscopy was subsequently employed to investigate the subcellular localization of BAG6 in *Rnf126*^*+/+*^ and *Rnf126*^−/−^ testes. In *Rnf126*^*+/+*^ samples, BAG6 displayed a distinct and well-defined distribution pattern aligned with active phases of spermatogenesis. Conversely, in *Rnf126*^−/−^ testes, BAG6 localization was significantly diminished or entirely absent, indicating a reliance on RNF126 for its proper spatial distribution within the spermatogenic microenvironment (Fig. 10C). BAG6 is a known inhibitor of apoptosis and a binding partner of BCL2. To assess whether RNF126 influences BCL2 expression, immunostaining for BCL2 was performed on testicular tissues from *Rnf126*^*+/+*^ and *Rnf126*^−/−^ mice. In *Rnf126*^*+/+*^ testes, BCL2 exhibited robust expression, particularly in regions with abundant actively dividing germ cells. However, *Rnf126*^−/−^ testes showed markedly reduced BCL2 signals, suggesting that RNF126 may indirectly regulate BCL2 localization through its interaction with BAG6 (Fig. 10D). TUNEL staining further revealed a significant increase in apoptotic germ cells during spermatogenesis in *Rnf126*^−/−^ mice compared to *Rnf126*^*+/+*^ controls (Fig. 10E–G). The germinal epithelium of *Rnf126*-deficient is characterized by a loss of germ cells secondary to an increase in different developmental stages germ cell apoptosis that ultimately leads to a vesiculation of spermatogenic tubule. These findings underscore the pivotal role of RNF126 in modulating BAG6-dependent pathways to regulate germ cell survival and spermatogenesis.

## Discussion

Multiple morphological abnormalities of the flagella (MMAF) represent one of the principal causes of male infertility. MMAF manifests as various structural defects in the sperm tail, including shortened, bent, or twisted flagella, which impair the sperm’s motility and mechanical propulsion [33]. These abnormalities are frequently attributed to disruptions in the structure or function of microtubular components within the sperm flagella [34]. Mutations in genes involved in flagellar biogenesis, axonemal assembly, and ciliary function are known to contribute to MMAF. For example, alterations in genes such as SPATA7, DNAH1, DNAI1, and DNAJB11 have been associated with MMAF-related infertility. The growing list of such genes underscores the extensive genetic heterogeneity underlying MMAF, suggesting that additional genes linked to this condition remain to be identified [35, 36]. Understanding the genetic basis of MMAF not only facilitates accurate diagnosis but also paves the way for personalized treatments and interventions for couples experiencing unexplained infertility.

RNF126, as an E3 ubiquitin ligase, exerts its influence on sperm flagellum formation through precise substrate recognition, efficient ubiquitin transfer, and subsequent modulation of substrate protein function and fate. One of the primary functions of RNF126 as an E3 ubiquitin ligase is its ability to specifically recognize target substrates involved in sperm flagellum formation. This recognition is highly selective and based on the unique structural and biochemical features of the substrate proteins. The sperm flagellum is a highly specialized organelle critical for motility and successful fertilization. Its central structural component, the axoneme, is composed of a cylindrical array of microtubules arranged in the classical “9 + 2” configuration [37]. Ultrastructural analyses of sperm exhibiting the MMAF phenotype frequently reveal a range of defects, from subtle abnormalities to significant malformations in key flagellar components [38]. The RNF (Ring Finger) protein family, characterized by their conserved RING domains that confer E3 ubiquitin ligase activity, plays a pivotal role in diverse cellular processes [39]. Within the context of germline development and spermatogenesis, RNF proteins emerge as critical regulators, orchestrating multiple processes essential for male fertility. For instance, RNF8 facilitates histone ubiquitination at specific genomic regions, recruiting nucleosome assembly and chromatin remodeling complexes, which suppress gene expression on sex chromosomes [40]. RNF19A contributes to acrosome formation and the assembly of outer dense fibers and the sperm tail sheath [41]. RNF216 ensures normal meiotic progression by targeting protein kinase A (PKA) catalytic subunits for degradation [42], while RNF144B promotes human spermatogonial stem cell proliferation and inhibits apoptosis via the FCER2/NOTCH2/HES1 signaling pathway [43]. Due to their extensive roles in spermatogenesis, mutations or dysregulation of RNF proteins are directly implicated in male reproductive disorders such as idiopathic infertility and azoospermia. Investigating these proteins deepens the understanding of reproductive biology and offers translational opportunities for therapeutic interventions.

This study provides novel evidence linking reduced expression of RNF126 to oligoasthenoteratospermia (OAT syndrome), suggesting that sufficient RNF126 levels are crucial for normal sperm morphology, motility, and overall health. RNF126 was found to be highly expressed in the testes of male mice. Immunostaining of *Rnf126*^*+/+*^ mice indicated RNF126 expression beginning in steps 12 and 13 of spermatid development, followed by its localization to the sperm midpiece, where it likely contributes to flagellar assembly. At this stage, spermatids undergo critical morphological changes, including nuclear elongation and flagellar organization, which are essential for functional motility [44]. In contrast, *Rnf126*^−/−^ mice exhibited a classic MMAF phenotype, characterized by short, twisted, irregular, or absent flagella, accompanied by reduced sperm count and motility defects. Male *Rnf126*^−/−^ mice were sterile and displayed significantly reduced testicular size, underscoring the profound impact of RNF126 deficiency on reproductive physiology. These findings are consistent with those obtained in single cell sequencing by Liu et al. In addition, our findings are consistent with clinical evidence of decreased RNF126 expression in patients diagnosed with oligoasthenospermia of unknown origin. But there is a slight immediate difference between the two results. This could be due to the important discovery of Liu et al. was based on the analysis of single cell sequencing of conditional different Rnf126 variant mouse model, while our study was based on the phenotype analysis of RNF126 knockout mice. We fully verified and analyzed the phenotype of germ cell specific markers at different developmental stages and obtained corresponding results [15]. These findings highlight the central role of RNF126 in the transformation of spermatids into mature spermatozoa, encompassing acrosome assembly and flagellar development.

RNF126 is also implicated in regulating cell death pathways, potentially modulating signaling cascades essential for germ cell survival and elimination. Dysregulated apoptosis can result in excessive germ cell loss, impairing spermatogenesis and reducing sperm production. The interaction between RNF126 and BAG6, a BCL2-binding adapter protein, offers significant insights into the molecular dynamics of spermatogenesis [45, 46]. BAG6, also known as BAT3 (BAG family molecular chaperone regulator 6), is a multifunctional protein involved in processes such as protein quality control, stress response, and cell differentiation [47]. Its role in spermatogenesis is particularly critical during meiosis, a stage characterized by extensive cellular reorganization. Misfolded proteins generated during this period pose a threat to cell integrity and must be efficiently removed to prevent apoptosis. BAG6 facilitates the clearance of such proteins, ensuring the survival and proper progression of spermatocytes to spermatids [48]. Additionally, BAG6 influences cell fate decisions in germ cells by regulating the degradation of proteins that govern cell cycle checkpoints and apoptosis [49]. BAG6’s involvement extends to flagellar biogenesis, where it supports the transport and incorporation of essential proteins into the developing axoneme [50]. The abnormal germ cells phenotype caused by BAG6 gene knockout is consistent with the *Rnf126* knockout we observed. Therefore, we have more reason to believe that *Rnf126* participates in the regulation of spermatogenesis and germ cell development through interaction with BAG6. This complicated balance between RNF126 and BAG6 is essential for the survival and differentiation of germ cells into fully functional spermatozoa.

## Conclusion

In conclusion, the findings presented herein establish RNF126 as a critical regulator within the intricate processes of germ cell development and spermatogenesis. The data underscore the essential role of RNF126 in governing germ cell apoptosis and flagellar biogenesis, as demonstrated by the significant sperm depletion and severe MMAF phenotypes observed in *Rnf126*^−/−^ mice. Furthermore, the interaction between RNF126 and BAG6 underscores the intricate regulation of protein quality control and cellular survival mechanisms during spermatogenesis. Understanding the functional role of RNF126 not only deepens insights into the fundamental biology of male reproduction but also holds significant translational potential for addressing idiopathic male infertility and enhancing the efficacy of assisted reproductive technologies.

## Materials and methods

### Generation of *Rnf126*^−/−^ mice and genotyping

The C57BL/6 *Rnf126*^*+/−*^ mice were generated by Cyagen Biosciences. Exons 1–8 of *Rnf126* were cut by gene-edited CRISPR/Cas9 technology with the sequences sgRNA1: 5′-CAGAAGTACCGTCCCGGGCTGCGG-3′ and sgRNA2: 5′-TGTAAAGAAGACTATGCACTGGG-3′. The male founder *Rnf126*^+/−^ mice were crossed with female *Rnf126*^+/−^ C57BL/6 mice to obtain F1 *Rnf126*^−/−^ mice for experiments. The mouse genotypes were confirmed by PCR with:

Primer F1: (5′- GGAGTGTTTAGAGTGGGTTCTTGT -3′)

Primer R1:(5′-CTAGGTTAAGGTGGGAGTTGTGT-3′)

Primer R2: 5′-TATGTCCAAAGCTACGAGGAAGAA-3′

The products were *Rnf126*^+/+^ 744 bp and *Rnf126*^−/−^ 315 bp. The *Rnf126*^+/−^ has two bands of 315 bp and 744 bp. All animal experiments in the study complied with regulatory standards approved by the Institutional Animal Care and Use Committee of Sun Yat-sen University (No. 00235).

### Analysis of *Rnf126*/RNF126 expression by database

The expression level of *Rnf126* gene in various tissues of mice is referred to the NCBI database (https://www.ncbi.nlm.nih.gov/gene/70294). The expression level of RNF126 in THE HUMAN PROTEIN ATLAS (https://www.proteinatlas.org/).

### Fertility assessment

Male and female mice of different genotypes (*Rnf126*^+/+^ female & *Rnf126*^+/+^ male, *Rnf126*^+/−^ female & *Rnf126*^+/−^ male, *Rnf126*^+/−^ female & *Rnf126*^−/−^ male, *Rnf126*^+/−^ male, *Rnf126*^+/+^ female & *Rnf126*^−/−^ male) aged 2–3 months were mated for at least 3 months to test fertility. The litter sizes, sex ratios, and birth intervals of each group of males and females were recorded.

### Real-time quantitative reverse transcription-PCR

TRIzol reagents (Thermo Fisher, Cat: 15596026CN) were used to extract total RNA from various tissues of mice, and reverse transcription was performed using (R323-01, Vazyme). Subsequently, qPCR was performed with (Q221-01, Vazyme) on a qPCR machine (Applied Biosystems™ 7500, ThermoFisher).

The primers were as follows:

*Rnf126*-F: GCCAGTTTGCCTTTGGCATC

*Rnf126*-R: GCCAGTTTGCCTTTGGCATC.

### Western blot

Total protein lysates were prepared from various tissues of mice using RIPA buffer (Vazyme, cat: R323-01s) supplemented with protease inhibitors (CWBio, cat: CW2200S). A total of 20 μg of total protein was separated using 10–12% gradient SDS-PAGE gels and then transferred to PVDF membranes (Merck Millipore, Cat.: IPVH00010). The PVDF membranes were further blocked with 5% nonfat dry milk in 1× Tris-buffered saline with Tween buffer (1× TBST) for 1 h at room temperature and then incubated with primary antibodies overnight at 4 °C. After removal of the primary antibodies, the PVDF membranes were washed three times (10 min each) in 1× TBST. The PVDF membranes were then incubated with secondary antibodies for 1 h at room temperature. After washing three times (10 min each) in 1× TBST, the target proteins were visualized using the ECL Enhanced Kit (Abclonal, Cat.: RM00021). The primary and secondary antibodies used in this study were as follows: RNF126 (Abcam, ab234812), β-actin (Abclonal, AC038), GAPDH (AC002, Abclonal), goat anti-mouse IgG (AC011, Abclonal), BAG6 (Abcam, ab137076; 26417-1-AP), HRP goat anti-mouse IgG (AS066, Abclonal), HRP goat anti-rabbit IgG (AS014, Abclonal).

### Histological analysis

Testes or epididymis were fixed overnight in 4% paraformaldehyde (DF0135, DF0135, Leagene) at 4 °C. Fixed tissues were embedded in paraffin and sectioned. Testicular or epididymal sections were stained with hematoxylin and eosin (C0105S, Beyotime).

### Immunofluorescence

Antigen retrieval was performed by boiling paraffin sections of the testis in sodium citrate antigen repair solution (G1202, Servicebio) for 20 min. The sections were then washed three times (10 min each) in PBS, exposed to blocking buffer (100 μL normal goat serum + 900 PBS) for 1 h at room temperature, and incubated with primary antibodies overnight at 4 °C. After removal of primary antibodies, sections were washed three times (10 min each) in 1×TBST before incubation with secondary antibodies for 1 h at room temperature and counterstained with DAPI (ZS, ZLI-9557) in the dark. The sections were observed with a confocal microscope (Zeiss LSM 880). The primary and secondary antibodies used in this study were as follows: RNF126 (ab234812, Abcam; sc-376005, Santa Cruz), γH2AX (AMAB91346, Sigma), SOX9 (A19710, Abclonal), PLZF (SC-28391, Santa), STRA8(ab308124, Abcam), PHH3 (#53348S, Cell Signaling), Ki67 (ab15580, Abcam), SYCP3(ab97672, Abcam), SYCP1 (ab303520, Abcam), MDC1 (A12714, Abclonal), TOPBP1 (A5781, Abcamal), BRCA1 (AP0232, Abclonal), 53BP1 (ab175933, Abcam), BAG6 (Abcam, ab137076; 26417-1-AP), BCL2 (A19693, A20777, Abclonal), goat anti-rabbit IgG H&L (Alexa Fluor® 488) (ab150077, Abcam), goat anti-rabbit IgG H&L (Alexa Fluor® 594)(ab150080, Abcam).

### Spermatocyte spreading

Testes were removed from the mice at P21 and the tunica albuginea was removed. The seminiferous tubules were treated with hypotonic buffer (30 mM Tris, 17 mM trisodium citrate dihydrate, 50 mM sucrose, 5 mM ethylenediaminetetraacetic acid, 0.5 mM dithiothreitol, and 0.5 mM phenylmethylsulfonyl fluoride, pH = 8.2) for 1 h at 4 °C treated. The tubes were cut into fragments and suspended in 100 mM sucrose on slides coated with fresh fixative solution (1% PFA, 0.15% Triton X-100, 10 mM sodium borate, pH = 9.2). The slides were placed in a humidified chamber at 4 °C overnight and washed with 0.4% Photo-Flo (Flo200, Kodak). For immunofluorescence, the procedure was the same as above for tissue immunofluorescence.

### Sperm staining

To observe sperm morphology, we stained the sperm with PNA, DAPI and anti-RNF126. The sperm was also stained with hematoxylin and eosin (H&E).

### Sperm count

One side of the cauda epididymis was removed from each mouse and cut into small pieces in 1 ml of 1x PBS. Sperm was released in an incubator at 37 °C for 30 min and inactivated at 58 °C for 15 min. Sperm counting was carried out using the hemocytometer.

### Scanning electron microscopy and transmission electron microscopy

Sperm was collected from cauda epididymis, washed three times with 1× phosphate-buffered saline (PBS) (pH 7.4) and fixed in 2.5% glutaraldehyde solution in 0.1 M PBS. The sperm were then fixed with 1% OSO4 for 1 h at RT and dehydrated sequentially in ethanol solutions (25%, 50%, 75%, and 95%). For scanning electron microscopy (SEM), samples were subjected to critical point drying and coated with gold/palladium for observation with a scanning electron microscope (ULTIMMAX 100, Oxford). For Transmission Electron Microscope (TEM), the samples were embedded in a resin and sliced. Ultrathin sections were stained with 2% uranyl acetate and lead citrate and observed using a transmission electron microscope (TECNAI G2 20 TWIN, FEI).

### RNA-seq and data analysis

Total RNAs were isolated from testes using TRIzol reagent. RNA quality was measured using the Agilent 2100 bioanalyzer. The mRNA was purified from total RNAs using poly-T oligo-linked magnetic beads and used to prepare the cDNA library. cDNA library preparation was evaluated using AMPure XP beads. Raw data was processed and calculated. All subsequent analyzes were based on high-quality data. Differential expression analysis (DEGs) of Rnf126+/+ and Rnf126−/− testis (three biological replicates per condition) was analyzed using DESeq2. The resulting *p* values were adjusted using the Benjamini and Hochberg approach to control the false discovery rate. Genes with an adjusted *p* value (*p*adj) < 0.05 found by DESeq2 were assigned as differentially expressed. The ClusterProfiler R package was used to test statistical enrichment of differential expression genes in Gene Ontology (GO).

### Immunoprecipitation and mass spectrometry (IP-MS) and co‐immunoprecipitation (Co-IP)

Testes from mice were homogenized in 800 μl of lysis buffer (25 mM Tris, 150 mM NaCl, 1 mM EDTA, 1% NP40, pH 7.4 and protease inhibitor cocktail). The lysate supernatant is obtained after ultrasonication and centrifugation at 12,000 rpm for 15 min at 4 °C. Protein concentration was measured using the BCA Protein Assay Kit (P0012, Beyotime). Proteins were immunoprecipitated with antibodies and the corresponding IgG (AC011, Abclonal) previously bound to protein A/G magnetic beads. After incubation at 4 °C overnight on an orbital shaker, beads were washed three times with NP40 buffer. Elution buffer (0.1 M glycine, pH 2.5) was added to the beads and incubated for 10 min at room temperature to elute the pull-down protein complexes from the beads. The eluents were collected for subsequent Western blotting and MS.

### Proteomics analysis

Protein from testis samples was extracted in lysis buffer (7 M urea, 2% SDS, and protease inhibitor cocktail) and digested with trypsin (V5280, Promega) for 16 h at 37 °C. The resulting peptide mixture was then redissolved in 20 mM aqueous ammonium formate (pH 10.0) and subjected to high pH separation on a reversed-phase column (BEH C18, Waters). The peptides were then redissolved in 30 μL of 0.1% formic acid water and then analyzed by LC tandem mass spectrometry (LC/MS) (Q ExactiveTM, Thermo). The raw LC/MS data were processed using Proteome Discoverer 2.2. Proteins differentially expressed in the two groups (DEPs) were subjected to trend analysis. KOGs, GO, KEGG and IPR analyzes were also performed.

### Statistical analysis

At least three independent assays were performed in triplicate. Values are presented as mean ± standard deviation (S.D.). Statistical analysis was performed using Student’s *t* test. Statistical significance was expressed as *p < 0.05, **p < 0.01; and ***p < 0.001. Statistical analysis in this study was performed using SPSS 25.0.

## Supplementary information

Figure S1

Figure S1

Full uncropped Gels and Blots image

## Data Availability

All the data are contained in the manuscript.
