# Supplementary material for: Absence of Rnf126 causes male infertility with multiple morphological abnormalities of the sperm flagella
Source: Cell Death Discov. 2025 May 23;11:251. doi: 10.1038/s41420-025-02432-w (PMC12102401; doi:10.1038/s41420-025-02432-w)
Supplement: Supplementary file 2 — Figure S1 [file 41420_2025_2432_MOESM2_ESM.docx]

**Figure S1. Expression and functional of RNF126 in *Rnf126^+/+^* and *Rnf126*^-/-^**

A. The human protein atlas database analyzed the localization of RNF126 in human testicular tissue.

B. The expression of RNF126 in germ cell deficient and germ cell present *Rnf126*^-/-^ mice testicular tissue sections. Scale bar = 50 μm.

C. Hematoxylin and eosin staining analysis of the morphology of the head and tail of the epididymis in *Rnf126^+/+^* and *Rnf126*^-/-^ mice. Scale bar = 500 μm.

D. Percentage of abnormal sperm in *Rnf126^+/+^* and *Rnf126*^-/-^ mice. Data are presented as average percentage, n=3 mice for each group, and 100 tubules were counted for each mouse.
