# Supplementary figures and images for: Absence of Rnf126 causes male infertility with multiple morphological abnormalities of the sperm flagella
Source: Cell Death Discov. 2025 May 23;11:251. doi: 10.1038/s41420-025-02432-w (PMC12102401; doi:10.1038/s41420-025-02432-w)

Figure 1D

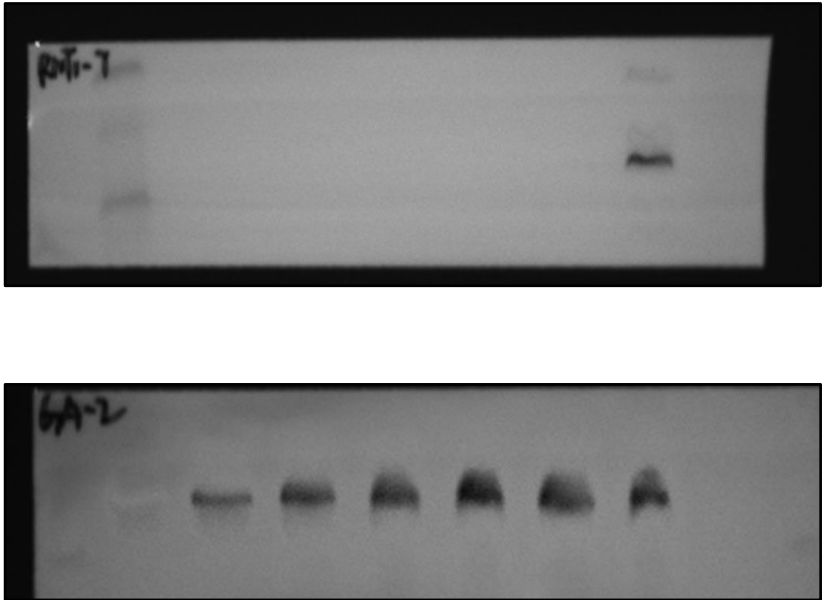

Figure 1F

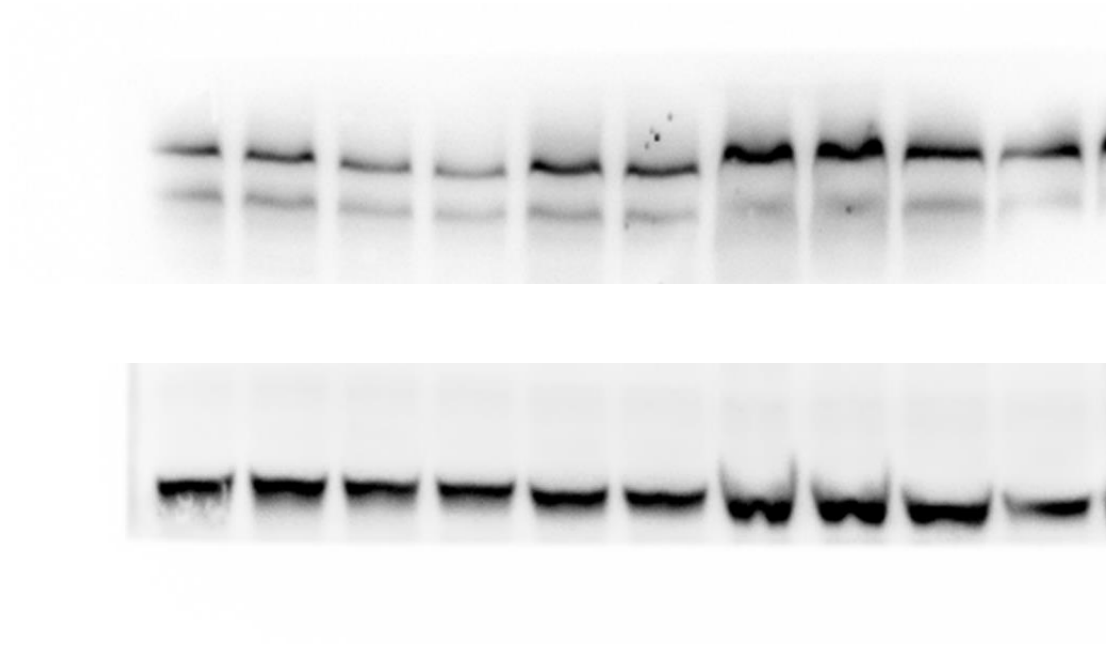

Figure 1F-Change

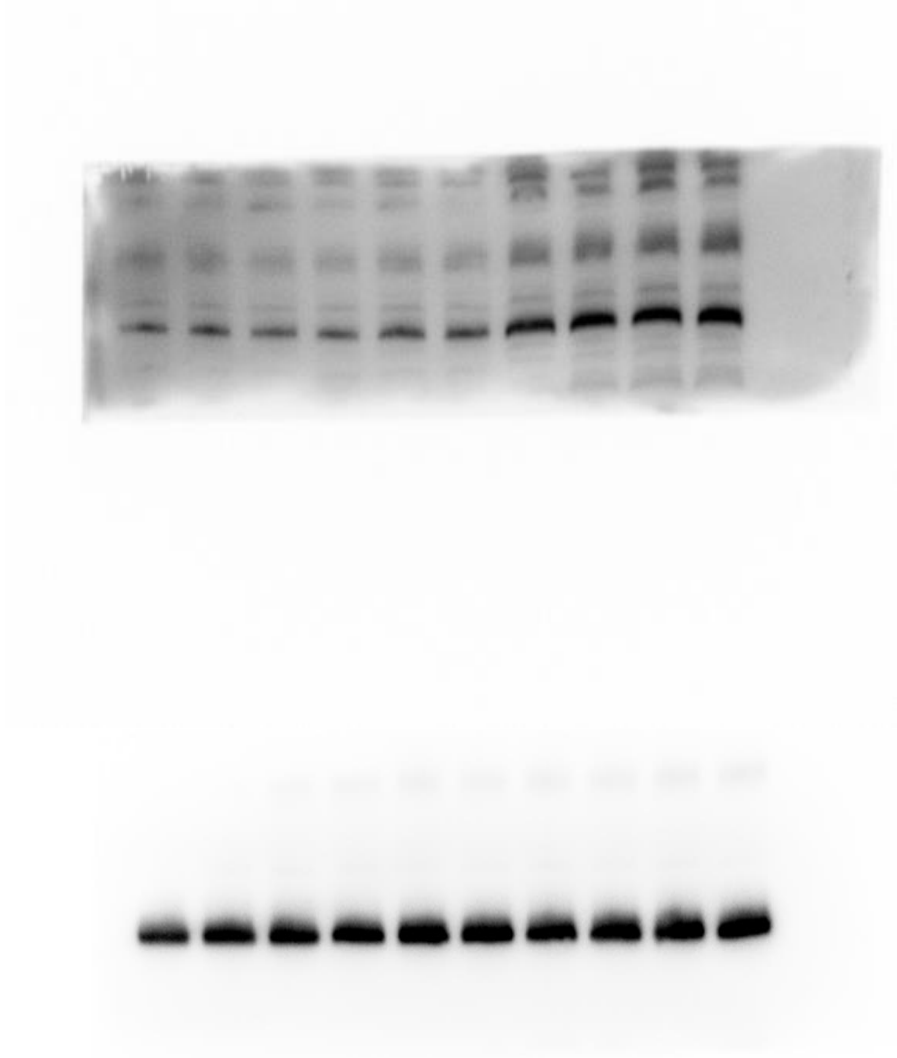

Figure 2C

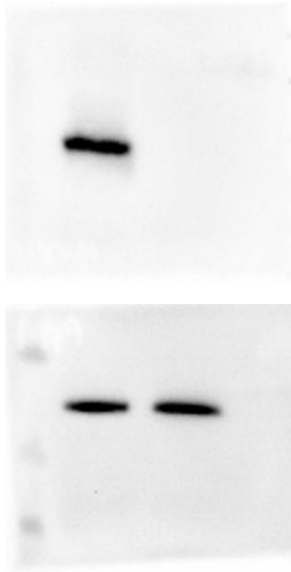

Figure 10A

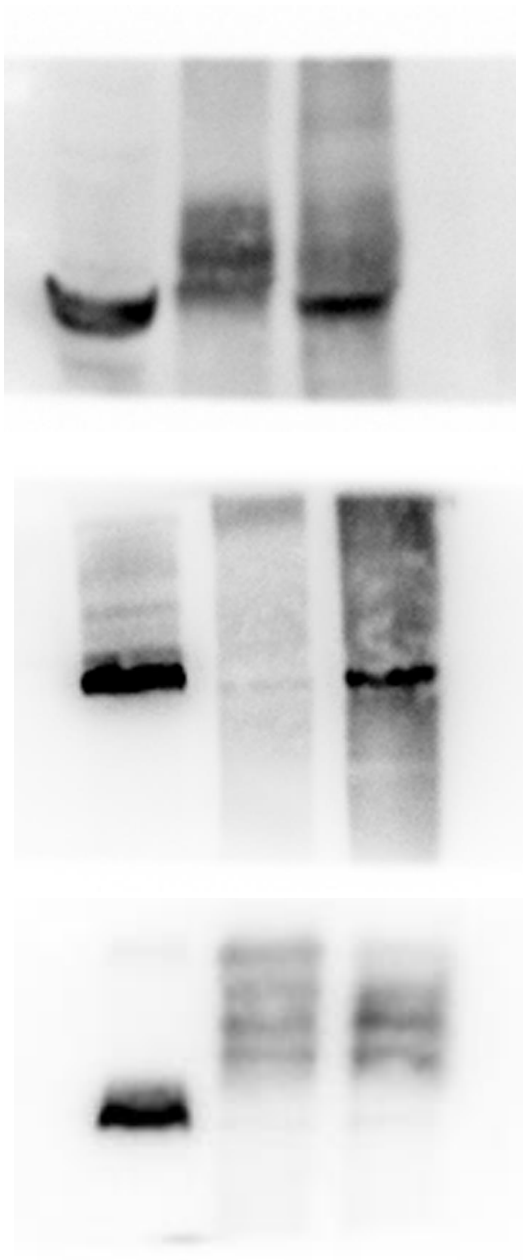

Figure 10B

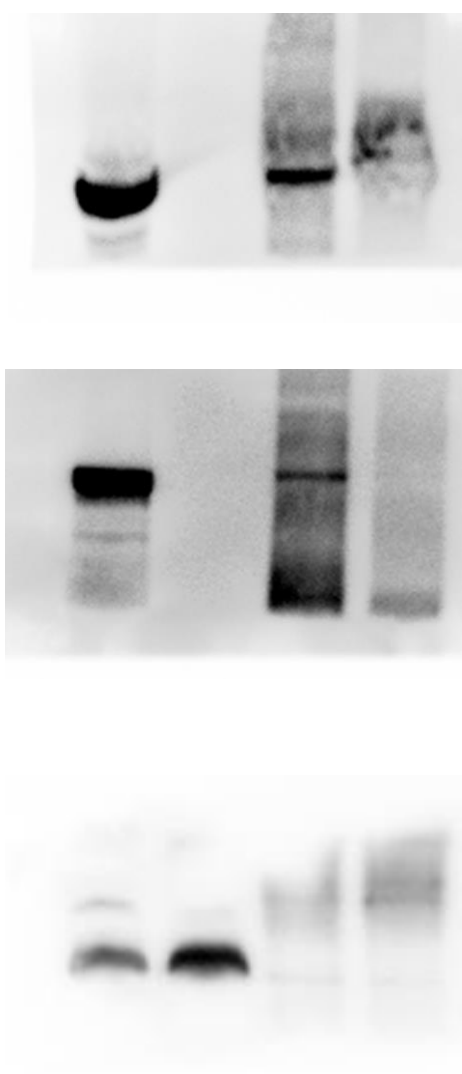

Supplement: Supplementary file 3 — Full uncropped Gels and Blots image [file 41420_2025_2432_MOESM3_ESM.pdf]
