# Supplementary material for: Absence of Rnf126 causes male infertility with multiple morphological abnormalities of the sperm flagella
Source: Cell Death Discov. 2025 May 23;11:251. doi: 10.1038/s41420-025-02432-w (PMC12102401; doi:10.1038/s41420-025-02432-w)
Supplement: Supplementary file 1 — Figure S1 [file 41420_2025_2432_MOESM1_ESM.pdf]

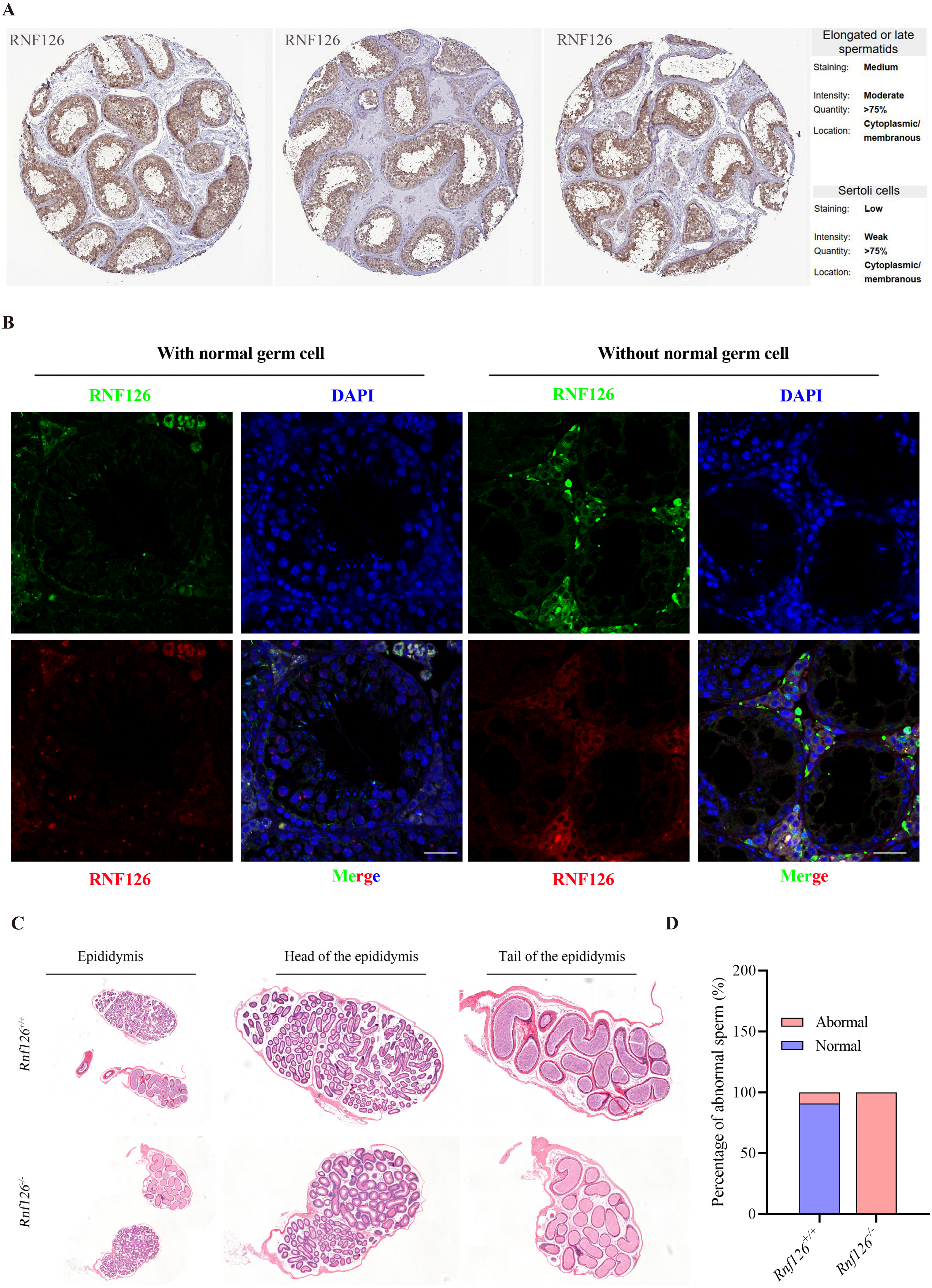
A
RNF126
RNF126
RNF126
Elongated or late spermatids
Staining: Medium
Intensity: Moderate
Quantity: >75%
Location: Cytoplasmic/ membranous
Sertoli cells
Staining: Low
Intensity: Weak
Quantity: >75%
Location: Cytoplasmic/ membranous
B
With normal germ cell
Without normal germ cell
RNF126
DAPI
RNF126
DAPI
RNF126
Merge
RNF126
Merge
C
Epididymis
Head of the epididymis
Tail of the epididymis
Rnf126+/+
Rnf126-/-
D
Percentage of abnormal sperm (%)
200
150
100
50
0
Abormal
Normal
Rnf126+/+
Rnf126-/-
